# A Study of Modified Infotaxis Algorithms in 2D and 3D Turbulent Environments

**DOI:** 10.1155/2020/4159241

**Published:** 2020-08-25

**Authors:** Shurui Fan, Dongxia Hao, Xudong Sun, Yusuf Mohamed Sultan, Zirui Li, Kewen Xia

**Affiliations:** Tianjin Key Laboratory of Electronic Materials Devices, School of Electronic and Information Engineering, Hebei University of Technology, Tianjin 300401, China

## Abstract

Emergency response to hazardous gases in the environment is an important research field in environmental monitoring. In recent years, with the rapid development of sensor technology and mobile device technology, more autonomous search algorithms for hazardous gas emission sources are proposed in uncertain environment, which can avoid emergency personnel from contacting hazardous gas in a short distance. Infotaxis is an autonomous search strategy without a concentration gradient, which uses scattered sensor data to track the location of the release source in turbulent environment. This paper optimizes the imbalance of exploitation and exploration in the reward function of Infotaxis algorithm and proposes a mobile strategy for the three-dimensional scene. In two-dimensional and three-dimensional scenes, the average steps of search tasks are used as the evaluation criteria to analyze the information trend algorithm combined with different reward functions and mobile strategies. The results show that the balance between the exploitation item and exploration item of the reward function proposed in this paper is better than that of the reward function in the Infotaxis algorithm, no matter in the two-dimensional scenes or in the three-dimensional scenes.

## 1. Introduction

With the development of gas sensor technology and mobile device technology, more and more researchers are facing gas-related research. Tracking the weak information released by the releasing sources in nature and human society through the olfactory tracking device can be regarded as the optimization problem of dynamic multiparameter function. Olfactory searchers can be used in areas related to the gas releases, such as toxic gas detection and location [1], rescue and relief [2], explosives detection and location [3] air pollution source tracking [4], and fire accident [5].

According to the search mode, the release source search algorithm can be divided into three categories: concentration tropism [6, 7], wind tropism [8–10], and information tropism [11]. According to the number of robots, it can be divided into multirobot cooperative search method [12, 13]and single-robot search method. Common multirobot collaboration algorithms include grey wolf algorithm [14], particle swarm optimization algorithm [15], bee colony algorithm [16], and so on. According to the search area, it can be divided into two-dimensional scene search algorithm [17] and three-dimensional scene search algorithm [18–20].

Concentration tropism algorithm and wind tropism algorithm are early olfactory localization algorithms inspired by biological behaviour [21–22], which have a long search path according to the evolution of predation and mate seeking behaviour. In recent years, there are also release source tracking algorithms that combine bioheuristic algorithm with vision [23, 24]. However, concentration tendency algorithms, such as the blue crab algorithm [22], have a patch and discontinuous particle distribution of the release source in a turbulent environment with a high Reynolds number, and the concentration gradient tracked by the mobile searcher is not reliable. Due to the complexity of plumes, it is important to collect as much information as possible. Wind tropism algorithms [8] are mostly applied to expensive commercial anemometers. Information tropism refers to an autonomous search algorithm based on information gain. This algorithm was first proposed by Vergassola et al. [11] in 2007, which provides a new way to solve the tracking problem of release sources. Compared with other kinds of autonomous search algorithms, when the concentration information is sporadic and sparse, even if the threshold value of the sensor carried by the searcher cannot be reached, the mobile searcher can well complete the task of autonomous search. Since then, more and more research studies have been conducted on information orientation algorithm [25, 26].

Masson [27] proposed a search algorithm in the two-dmensional limited environment perception scene, which uses free energy as the information acquisition metric of mobile tracking devices. Rodriguez et al. [28] applied the Infotaxis algorithm to finite two-dimensional lattices with different geometric shapes. In 2014, Ristic et al. [29] proposed an autonomous search algorithm in the two-dimensional scene with obstacles, which is called enfotaxis. Ristic et al. [30] used Rényi divergence as information acquisition to measure the autonomous search method based on sequential Monte Carlo combination in the two-dimensional scene and analyzed it in combination with various reward functions. Hutchinson et al. [31] proposed an information destination algorithm based on Kullback–Leibler divergence as a measure of information acquisition and simulated it in real environment. The results show that the algorithm has good search performance in real environment. Rodríguez et al. [32] proposed a blind information algorithm in the two-dimensional scene and compared the search success rate with the traditional Infotaxis algorithm under a variety of fuzzy environment conditions. Ruddick et al. [33] extended the Infotaxis algorithm to three-dimensional space for application and applied the algorithm to the actual environment for verification. In 2019, Song et al. [34] proposed the minimum free energy search method based on the combination of entropy and potential energy in Monte Carlo. Hai-Feng et al. [35] proposed an underwater chemical plume tracking system, which used the combination of partially observable Markov decision algorithm and artificial potential field algorithm to construct the underwater source probability map. Park and Oh [13] conducted a large number of simulation analyses by applying a sequential Monte Carlo Infotaxis algorithm with particle filter and combined them with a variety of reward functions in a two-dimensional scene by using a multirobot.

Although the literatures mentioned above have improved the Infotaxis algorithm to some extent, they are only improved in a single scene (two-dimensional or three-dimensional scenes), and there is no comparative analysis on the universality of different decision functions and mobile strategies in two-dimensional and three-dimensional scenes. Therefore, this paper proposes a decision function for Infotaxis algorithm and analyzes the search performance of the new modified Infotaxis algorithms in two-dimensional and three-dimensional scenes using different mobile strategies as well as proposes a new mobile strategy for three-dimensional search scenes.

## 2. Infotaxis Algorithm

**Figure 1:**
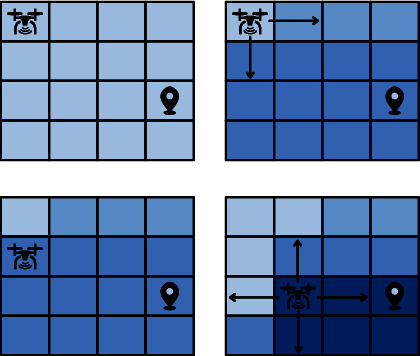
Infotaxis algorithm.

Infotaxis algorithm can be divided into three steps: information state, reward functions, and mobile strategy. The search area is gridded. The algorithm initially assumes that each square has the same probability of releasing the source. The mobile searcher evaluates the value of each alternative direction reward function in the mobile strategy and chooses the direction of the maximum information entropy drop as direction, as shown in Figure 1. The mobile searcher divides and updates the probability distribution of each square with each step forward. The darker the square is, the higher the probability that the square is the source of the release. With the decrease of information entropy, the mobile searcher will get closer to the release source.

### 2.1. Gas Diffusion Model

**Figure 2:**
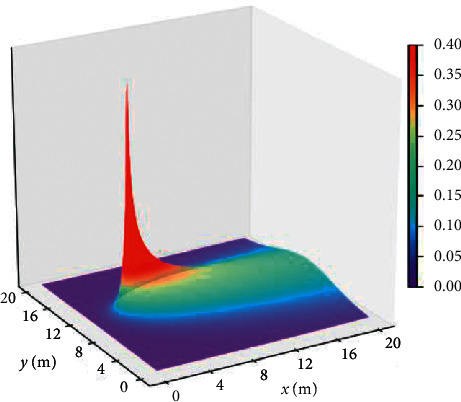
Two-dimensional turbulent diffusion model.

The premise of the Infotaxis algorithm is that the mobile searcher is able to predict the concentration of particles released by the emission source around the location, which is to predict the concentration of particles released by the emission source everywhere in the space through the gas diffusion model. General gas diffusion model satisfies the following equation:

(1) $$\begin{array}{c} 0=V\nabla C\left(r\left|r_{s}\right.\right)+D\Delta \;C\left(r\left|r_{s}\right.\right)-\frac{1}{\tau }C\left(r\left|r_{s}\right.\right)+R\delta \left(r-r_{s}\right), \end{array}$$

where *V*represents the average wind speed in unit m/s; *D* refers to the effective isotropic diffusion coefficient in the turbulence model, in unit m^2^/s; *R* refers to the release rate of the release source; *τ* refers to the average life of particles released by the releasing source, in unit s; *δ*(*r* − *r*_*s*_) is Kronecker delta; and *C*(*r*|*r*_*s*_) refers to the particle concentration at *r* if the release source is at *r*_s_. After *C*(*r*|*r*_*s*_) is processed, the two-dimensional turbulent diffusion model is transformed into equation (2), and the three-dimensional turbulent diffusion model *C*(*r*|*r*_*s*_) is transformed into equation (3).

(2) $$\begin{array}{c} C\left(r\left|r_{s}\right.\right)=\frac{R}{2\pi \;D}e^{\left(-\left(x-x_{s}\right)V/2\;D\right)}K_{0}\frac{\left|r-r_{s}\right|}{\lambda },\;\lambda =\sqrt{\frac{D\tau }{1+\left(V^{2}\tau /4\;D\right)}}, \end{array}$$

where *K*_0_ is the Bessel function with zero order modification; and |*r* − *r*_*s*_|is the linear distance between the current position of the mobile searcher and the released source. The diffusion model is the expression of the classical turbulent diffusion model in two-dimensional space. The simulation diagram is shown in Figure 2. The coordinate of the release source with the release intensity of 2Hz is(4,11), the wind with the average velocity of 1m/sblows along the positive direction of *x*axis, the effective diffusion coefficient of isotropy is 0.6m^2^/s, and the life of a particle is 500s.

(3) $$\begin{array}{c} C\left(r\left|r_{s}\right.\right)=\frac{R}{4\pi \;D\left|r-r_{s}\right|}e^{\left(-\left(x-x_{s}\right)V/2\;D\right)}e^{\left(-\left|r-r_{s}\right|/\lambda \right)},\;\lambda =\sqrt{\frac{D\tau }{1+\left(4^{2}\tau /4\;D\right)}}. \end{array}$$

**Figure 3:**
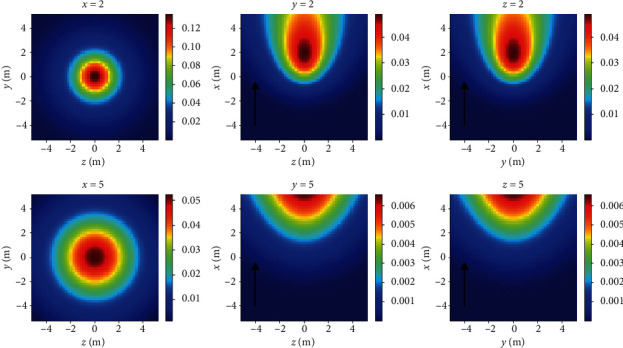
Three-dimensional turbulent diffusion model.

The diffusion model is the expression of the classical turbulent diffusion model in three-dimensional space, reflecting the relationship between the particle concentration of the releasing source and some variables in three-dimensional space.  Figure 3 shows the three-dimensional simulation section diagram of the model, and the black arrow represents the wind direction. The simulation range is a three-dimensional space of 10m × 10m × 10m, which is divided into 50 × 50 × 50 cubes.The coordinate of the release source is (0,0,0); other parameters are the same as Figure 2.

### 2.2. Information Status

The information state is expressed by a probability distribution *P*(*r*_*s*_*|L*_1:*k*_) when determining the location of the release source, and *r*_*s*_ refers to the location of the leakage source. *L*_1:*k*_ = {*Z*_*i*_(*r*_*i*_)}_1≤*i*≤*k*_ refers to the collection of all measured values of the mobile tracking equipment from the beginning to the time *k*. Using Bayesian framework to update posterior probability distribution, the updating process is shown in the following equation:

(4) $$\begin{array}{c} P\left(r_{s}\left|L_{1:k+1}\right.\right)=\frac{g\left(z_{k+1}\left(r_{k+1}\right)\left|r_{s}\right.\right)P\left(r_{s}\left|L_{1:k}\right.\right)}{P\left(z_{k+1}\left(r_{k+1}\right)\left|L_{1:k}\right.\right)}, \end{array}$$

where *P*(*z*_*k*+1_(*r*_*k*+1_)|*L*_1:*k*_) = ∫*g*(*z*_*k*+1_(*r*_*k*+1_)|*r*_*s*_)*P*(*r*_*s*_|*L*_1:*k*_)d*r*_*s*_is the normalization constant. Because the events of particles released by release source detected and particles released by release source not detected are independent and obey Poisson distribution, the number of plume detection between *k* − 1 and *k* time is expressed by *g*(*z*(*r*)|*r*_*s*_) = (*μ*^*z*^/*z*)*e*^−*μ*^, where *μ* = *R*(*r*|*r*_*s*_)*β*. *β* is the time interval of detection. *R*(*r*|*r*_*s*_) is given by the Smoluchowski's arguments to describe the probability that the mobile searcher with radius of *a* meter detects the particles released by the release source (equation (5)). The expressions of this equation in two-dimensional and three-dimensional environments are, respectively, equations (6) and (7). In particular, in a two-dimensional environment, the value of *λ* must be larger than that of *a*.

(5) $$\begin{array}{c} R\left(r\left|r_{s}\right.\right)=\frac{2\pi \;DC\left(r\left|r_{s}\right.\right)}{\text{In}\left(\lambda /a\right)}, \end{array}$$

(6) $$\begin{array}{c} R\left(r\left|r_{s}\right.\right)=\frac{R}{\text{In}\left(\lambda /a\right)}e^{\left(\left(x-x_{s}\right)V/2\;D\right)}K_{0}\left(\frac{\left|r-r_{s}\right|}{\lambda }\right), \end{array}$$

(7) $$\begin{array}{c} R\left(r\left|r_{s}\right.\right)=\frac{aR}{\left|r-r_{s}\right|}e^{\left(\left(x-x_{s}\right)V/2\;D\right)}e^{\left(-\left|r-r_{s}\right|/\lambda \right)}. \end{array}$$

### 2.3. Reward Function

Reward function is a criterion used to evaluate information measurement, which determines how the mobile searcher moves. The two traditional decision functions and the reward function proposed in this paper are compared.

#### 2.3.1. Reward Function of Infotaxis

Information entropy can be used to describe the uncertainty of the location of the released source. The higher the uncertainty, the higher the entropy, which is the core idea of the Infotaxis algorithm. The algorithm updates the probability map of release source based on Bayesian equation and calculates the change of information entropy according to the probability to determine the moving direction of the mobile searcher. The expression of information entropy at time *t* is described in the following equation:

(8) $$\begin{array}{c} S_{t}=-\sum P\left(r_{s}\left|L_{1:k}\right.\right)\log\;P_{t}\left(r_{s}\left|L_{1:k}\right.\right). \end{array}$$

The information entropy of the alternative moving position at the next moment is predicted, and the direction in which the information entropy decreases the fastest is chosen as the starting position of the next moving position. The subtraction equation of information entropy is shown in the following equation:

(9) $$\begin{array}{c} \Delta S_{\text{full}}=P_{s\_\mathrm{found}}\left(r_{s}\left|L_{1:k}\right.\right)\left(-S_{t}\right)+P_{s\_\mathrm{not\_found}}\left(r_{s}\left|L_{1:k}\right.\right)\Delta S_{s\_\text{not}\_\mathrm{found}}, \end{array}$$

where *P*_*s*_found_ refers to the probability of finding the release source on the probability map; *P*_*s*_not_found_ refers to the probability sum of not finding the release source on the probability map; and −*S*_*t*_ refers to the decrease of information entropy when the release source is found. When the release source is found in an ideal situation, the information entropy is 0. Δ*S*_*s*_not_found_=∑_*i*=0_^*k*^(*P*(hit=*i*)Δ*S*_*i*_) refers to the decrease of information entropy without finding the release source.

#### 2.3.2. Reward Function of InfotaxisII

When the searcher performs the search task, *P*_*s*_found_ in the exploitation item of equation (9) is meaningful only when the release source *r*_*s*_ matches the node in the searched area [30]. Therefore, the first item in equation (9) is discarded directly, making *P*_*s*_found_ = 0. Get the decision function:

(10) $$\begin{array}{c} \Delta S_{\text{full}}=P_{s\_\text{not}\_\text{found}}\left(r_{s}\left|L_{1:k}\right.\right)\Delta S_{s\_\text{not}\_\text{found}}. \end{array}$$

#### 2.3.3. Reward Function of Sinfotaxis

(11) $$\begin{array}{c} \Delta S_{\text{full}}=\left(\sum_{\left\|r-r_{s}\right\|\leq L_{th}}P\left(r_{s}\left|L_{1:k}\right.\right)\right)\left(-S_{t}\right)+\left(\sum_{\left\|r-r_{s}\right\|\leq L_{th}}P\left(r_{s}\left|L_{1:k}\right.\right)\right)\Delta S_{s\_\text{not}\_\text{found}}. \end{array}$$

As shown in equation (11), ‖*r* − *r*_*s*_‖is the distance between the location of the current searcher and the assumed release source *r*_*s*_. *L*_*th*_ is the threshold value that considers the distance to find the release source. In Infotaxis algorithm, the first item of reward function is exploitation, and the second item is exploration. The role of exploitation is to determine the location of the source by using the information obtained so that the explorer is more inclined to move in the direction of the source. The role of exploration is to explore more location areas for the searcher to increase the certainty of the location of the source. Equation (11) increases the role of exploitation, limits the exploration of the searcher, reduces the unnecessary exploration of the unknown area by the searcher, increases the guidance role of the source, and makes the searcher to explore in the direction of releasing the source.

### 2.4. Mobile Strategy

When the mobile searcher judges each alternative moving direction through the reward function, the next moving direction is selected according to the evaluation result. The set of alternative directions of movement is called mobile strategy. For two-dimensional search, there are at least four alternative directions {(*x* + Δ*l*, *y*), (*x* − Δ*l*, *y*), (*x*, *y* + Δ*l*), (*x*, *y* − Δ*l*)} when the mobile searcher is at *r*(*x*, *y*); for three-dimensional search, there are at least six alternative directions {(*x* + Δ*l*, *y*, *z*), (*x* − Δ*l*, *y*, *z*), (*x*, *y* + Δ*l*, *z*), (*x*, *y* − Δ*l*, *z*), (*x*, *y*, *z* + Δ*l*), (*x*, *y*, *z* − Δ*l*)} when the mobile searcher is at *r*(*x*, *y*, *z*). The reasonable choice of movement mode can effectively reduce the length of the search path of the mobile searcher so that the mobile searcher can locate the release source more quickly. Therefore, this paper will expand the mobile orientation in two-dimensional and three-dimensional scenes and discuss the performance of several Infotaxis algorithms after the increase of mobile orientation.

## 3. Infotaxis Algorithms for Two-Dimensional Scene

### 3.1. Mobile Strategy in Two-Dimensional Scene

**Figure 4:**
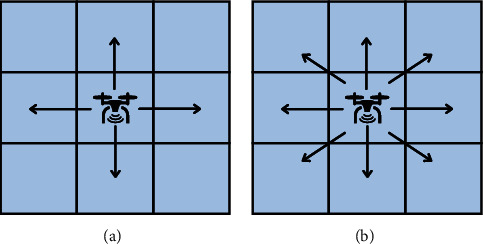
Mobile strategy in two-dimensional scene: (a) a mobile strategy with four alternative directions; (b) a mobile strategy with eight alternative directions.

Most of the previous literatures have carried out a lot of simulation analysis, but there are not many Infotaxis algorithms with different combinations of mobile strategies and reward functions. Different reward functions and different mobile strategies are combined to form the new Infotaxis algorithms, which will produce different search performances. Therefore, in the two-dimensional scene, this paper considers two groups of acceptable mobile strategies, as shown in Figure 4. The location of the mobile search device is marked by the UAV icon, and the black arrow points to the alternative direction of movement of the mobile strategy.

### 3.2. Simulation Analysis of Infotaxis Algorithm in Two-Dimensional Scene

**Figure 5:**
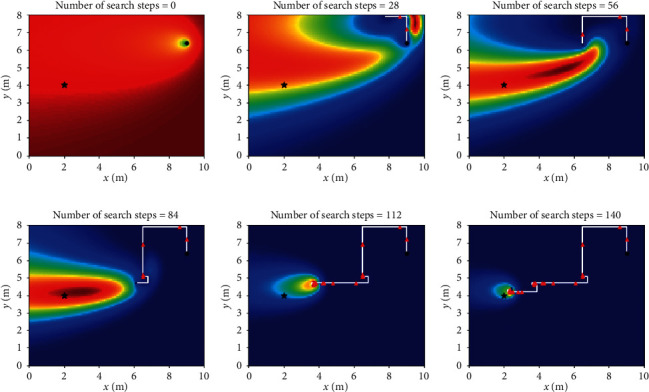
Infotaxis_4 two-dimensional simulation route.

**Figure 6:**
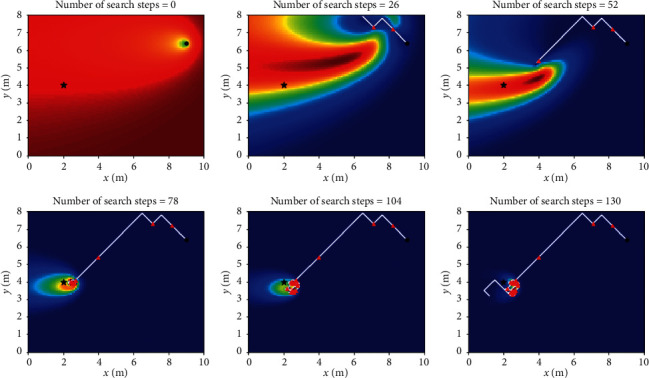
Infotaxis_8 two-dimensional simulation route.

**Figure 7:**
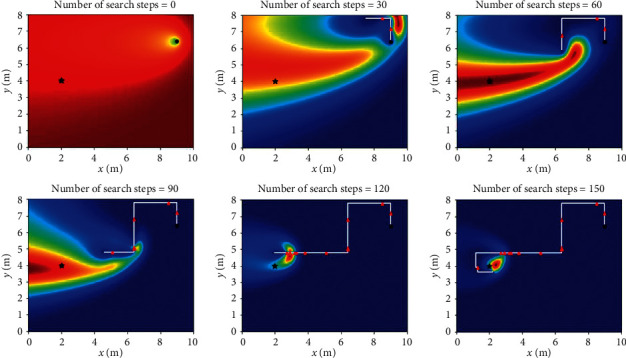
InfotaxisII_4 two-dimensional simulation route.

**Figure 8:**
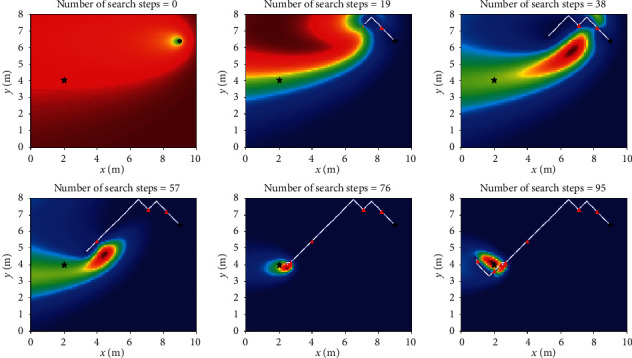
InfotaxisII_8 two-dimensional simulation route.

**Figure 9:**
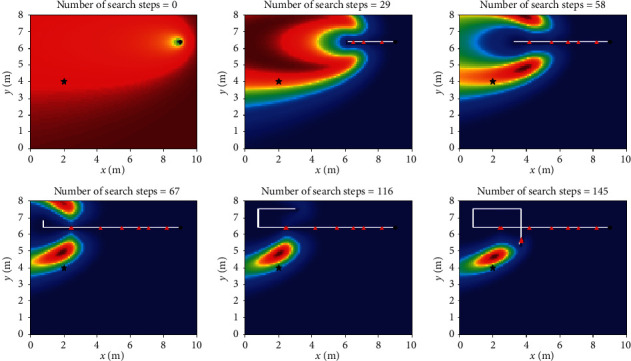
Sinfotaxis_4 two-dimensional simulation route.

**Figure 10:**
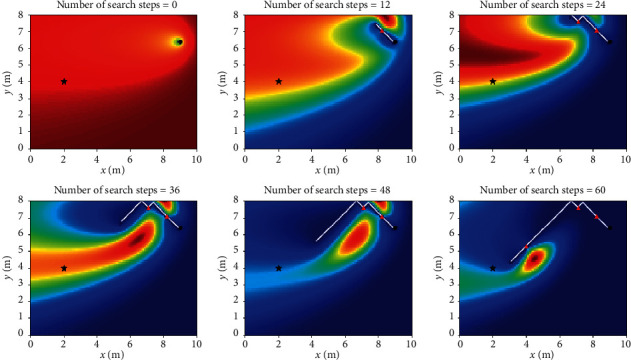
Sinfotaxis_8 two-dimensional simulation route.

Let *L*_th_ = *α*Δ*l*, and Δ*l* is the size of fine granularity of simulation environment, where *α* cannot be negative; when 0 ≤ *α* < 1, Sinfotaxis is equal to Infotaxis. In the two-dimensional scene, the distance threshold of Sinfotaxis is *L*_th_ = Δ*l*. Two mobile strategies mentioned in Figure 4 and three reward functions mentioned above are, respectively, simulated and analyzed. The six combined Infotaxis algorithms are as follows: Figure 5 shows the simulation diagram of traditional Infotaxis adopting the mobile mode of Figure 4(a), and Figure 6 shows the simulation diagram of traditional Infotaxis adopting the mobile mode of Figure 4(b).  Figure 7 shows the simulation diagram of InfotaxisII adopting the mobile mode of Figure 4(a), and Figure 8 shows the simulation diagram of InfotaxisII adopting the mobile mode of Figure 4(b).  Figure 9 shows the simulation diagram of Sinfotaxis adopting the mobile mode of Figure 4(a) when *L*_th_ = Δ*l*, and Figure 10 shows the simulation diagram of Sinfotaxis adopting the mobile mode of Figure 4(b) when *L*_th_ = Δ*l*.

The simulation environment is a two-dimensional region of 10m × 8m, and the fine granularity of simulation is 0.1m × 0.1m. When the linear distance between the mobile searcher and the release source is no more than 0.2m, the mobile search device is considered to have located the leak source. In the simulation diagram, the black star is the location of the release source, with the coordinates of (2,4); the black dot is the starting location of the mobile search device, with the coordinates of (9,6.4); the white line is the search path of the mobile search device, and the red triangle represents the particle information released by the release source detected by the mobile search device at this location. The wind direction is positive along the *x*-axis, and the threshold of concentration detected by the mobile search device is 0.005. The wind speed is 1m/s. The existence time of the particle is *τ*=100*s*. The radius of the searcher is *a*=0.1m. The intensity of the release source is *R*=0.6Hz, and the diffusion coefficient is *D*=0.5m^2^/s.

The mobile searcher initially estimated the location information estimation map of the release source which satisfies the turbulence diffusion model. As can be seen from Figures 5–10, with the increase of search steps, the mobile searcher judges the source location information around the path through the searched path in the past. With the search continuing, the mobile searcher continuously estimates the release source location accurately and finally determines the location of the leak source. For mobile search equipment, the information around the searched path is mostly known, and the probability distribution of the release source in the unsearched area also satisfies the turbulence diffusion model to some extent.

**Figure 11:**
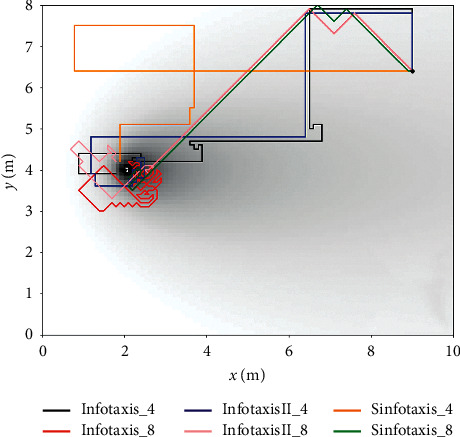
Search path of two-dimensional simulation.

Figure 11 shows the path process of six Infotaxis algorithms searching the release source in the same experimental environment. It can be seen from Figure 11 that all six Infotaxis algorithms can successfully locate the location of the release source. The total search steps of the six Infotaxis algorithms are 170 (Infotaxis_4),161 (Infotaxis_8), 157 (InfotaxisII_4), 116 (InfotaxisII_8), 174 (Sinfotaxis_4), and 77 (Sinfotaxis_8). It can be seen from Figure 11 that the traditional Infotaxis under the two mobile strategies and the InfotaxisII algorithm based on the four alternative mobile strategies are easy to make the search fall into local extremum, that is, the mobile search device will carry out multiple explorations in a small area. On the whole, the movement mode of the eight alternative positions in the move strategy has more advantages in the process of releasing source search. Because wind is complex and changeable in real environment, this advantage is more obvious when applying the Infotaxis based on eight alternative orientation move strategies.

**Figure 12:**
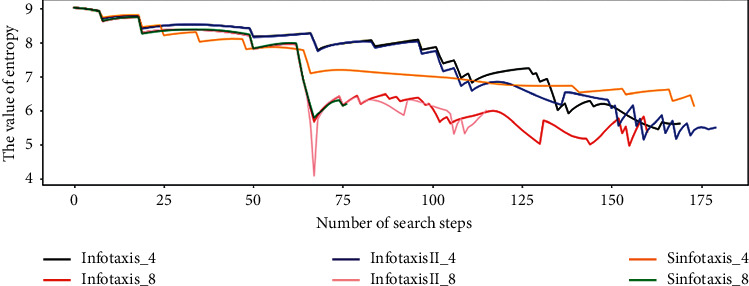
Entropy change of two-dimensional simulation.

Figure 12 shows the variation trend of entropy value of releasing source position during the search process of the six Infotaxis algorithms. When the steps of the six Infotaxis algorithms are less than 50, the degree of entropy decrease and the trend of entropy decrease are basically the same. When the six Infotaxis algorithms locate the released source, the final entropy is 5.6246 (Infotaxis_4), 5.3805 (Infotaxis_8), 5.5052 (InfotaxistII_4), 6.0027 (InfotaxisII_8),6.1376 (Sinfotaxis_4), and 6.1911 (Sinfotaxis_8). Figure 10 shows that the entropy value is not the minimum value of the entropy in the search process, when they locate the release source. So it is not advisable to take the entropy value as the search task stop condition in the two-dimensional scene. It can also be seen from Figure 12 that when the mobile searcher predicts and detects the particles released by the release source, the change of entropy will fluctuate; especially when the Infotaxis algorithm falls into a local extreme value, the entropy changing curve will fluctuate greatly. To further compare these six Infotaxis algorithms, this paper randomly selects different starting points and parameters (*R*, *D*, *V*)for 100 times of simulation and evaluates them through average steps. As shown in Table 1, it can be seen that Sinfotaxis_8 has good search performance in two-dimensional search.

## 4. Infotaxis Algorithms for Three-Dimensional Scene

### 4.1. Mobile Strategy in Three-Dimensional Scene

**Figure 13:**
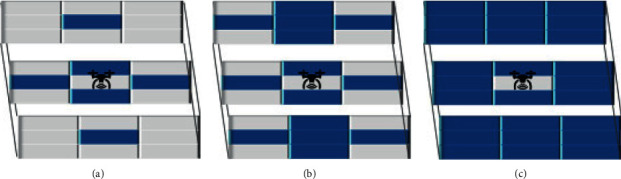
Mobile strategy in three-dimensional scene: (a) a mobile strategy with six alternative directions; (b) a mobile strategy with fourteen alternative directions; (c) a mobile strategy with twenty-six alternative directions.

When analyzing the performance of the Infotaxis algorithm in the three-dimensional scene, this paper proposed three kinds of mobile strategies combined with three kinds of reward functions. The cube of the three mobile strategies is shown in Figure 13, where the mobile searcher is located and marked with UAV icon. The cube marked in blue represents where the mobile searcher is likely to move at the next moment. Three kinds of mobile strategies are proposed according to *L*_th_ = *α*Δ*l*, selecting the probability range of *P*_*s*_found_ when *α* is different. When*α* = 1, the area is as shown in Figure 13(a), with 6 alternative moving directions. When$\alpha =\sqrt{2}$, the area is as shown in Figure 13(b), with 14 alternative moving directions. And when$\alpha =\sqrt{3}$, the area is as shown in Figure 13(c), with 26 alternative moving directions.

### 4.2. Simulation Analysis of Infotaxis Algorithm in Three-Dimensional Scene

In this paper, three thresholds were proposed for the Sinfotaxis algorithm in the three-dimensional scene: *L*_th_=Δ*l*, $L_{\text{th}}=\sqrt{2}\Delta l$, and $L_{\text{th}}=\sqrt{3}\Delta l$, named Sinfotaxis, SinfotaxisII, and SinfotaxisIII, respectively, which are combined with the Infotaxis and InfotaxisII proposed before to form five reward functions totally. The five reward functions are combined with the three mobile strategies in Figure 13 to form fifteen Infotaxis algorithms.

**Figure 14:**
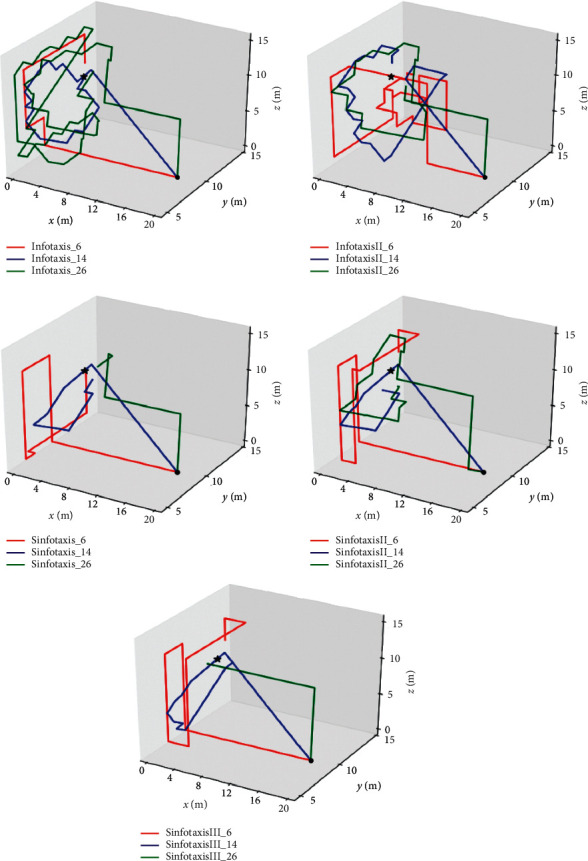
Track route of three-dimensional simulation.

The mobile searcher used in the three-dimensional scene reference is usually a small UAV (without considering the rotor's disturbance to the air flow). The radius of this UAV is generally *a*=0.2m. Considering that the search range of the three-dimensional scene in real scene is wider than the two-dimensional scene and the required fine granularity is not high, the scope of simulation space is set as three-dimensional scene of 20m × 10m × 15m with fine granularity of 1m × 1m × 1m. When the straight-line distance between the mobile search device and the release source is not greater than 1.7*m*, it is considered that the mobile search device has located the leak source. The wind direction is positive along the *x*-axis, the threshold value of concentration detected by the mobile search device is 0.005, the wind speed is 1m/s, the particle existence time is *τ*=500s, the release source intensity is *R*=5Hz, and the diffusion coefficient is *D*=0.6m^2^/s. The simulation roadmap is shown in Figure 14. The location of the release source is represented by a black star, and the black dot represents the starting point of the mobile searcher. The total number of search steps of the 15 information orientation algorithms is shown in Table 2.

Fifteen Infotaxis algorithms have successfully completed the search task. As can be seen from Figure 14, when the traditional Infotaxis is adopted in the three-dimensional scene, the mobile strategy with 14 alternative directions is relatively best. It has the minimum number of search steps, but there is no phenomenon of falling into local extreme values in the two-dimensional scene. Combined with Table 2, it can be concluded that in this simulation condition, the worst performance of the three mobile strategies in the three-dimensional scene is the mobile strategy (Figure 13(b)) with 6 alternative mobile directions. In terms of reward function, no matter which kind of mobile strategy is adopted, the performance of Sinfotaxis algorithm in the three-dimensional scene is the best among fifteen Infotaxis algorithms, which shows that when threshold value is *L*_th_ = Δ*l*, the exploitation and exploration in reward function reach a good balance. In contrast, InfotaxisII algorithm with six alternative moving directions is not suitable for tracking release source tracking events in the three-dimensional scene.

**Figure 15:**
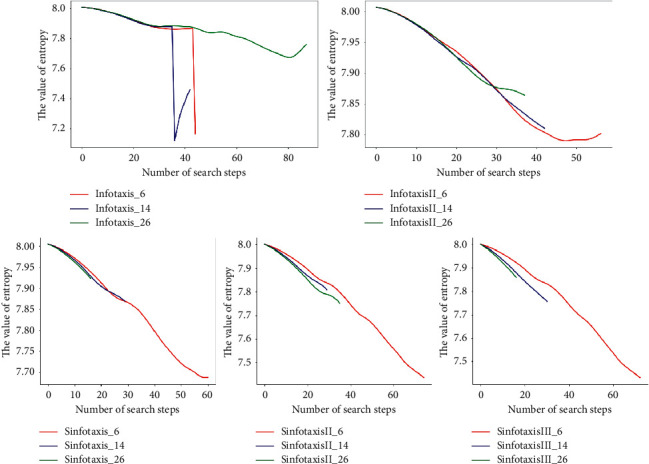
Entropy change of three-dimensional simulation.

Figure 15 shows the entropy changing curve of fifteen Infotaxis algorithms when performing search tasks in the three-dimensional scene. The ratio of the value of equation (7) in the three-dimensional scene to that of equation (6) in the two-dimensional scene is too small. Therefore, when using the Poisson distribution, the number of times the mobile searcher meets the particles released by the release source between its own position and the next position is basically zero, which leads to the change of the entropy value of the Infotaxis algorithm in the three-dimensional scene which is much more stable than that in the two-dimensional scene. In order to obtain more objective data, the fifteen Infotaxis algorithms proposed in this paper were simulated for 50 times when the starting point and parameters of the search were changed, and the average number of search steps in Table 3 was obtained. The results show that SinfotaxisIII_14 has fewer steps to locate the release source than other Infotaxis algorithms.

On the whole, the search path of the mobile strategy with 26 alternative directions is inclined to explore too many unknown areas, and it is not suitable for the event of three-dimensional scene tracking release source. The reason for this phenomenon is that the straight-line distance from each alternative direction to the mobile searcher is different in the mobile strategy with 26 alternative directions, which will cause a certain error. Although this error exists in the movement strategy with fourteen alternative directions, it is not enough to affect the performance of the Infotaxis algorithm.

## 5. Conclusions

In this paper, a variety of reward functions and mobile strategies for Infotaxis algorithms were discussed in two-dimensional and three-dimensional scenes. The simulation results show that Sinfotaxis_8 algorithm with 8 alternative movement directions has good search performance in the two-dimensional scene, and Sinfotaxis_14 algorithm with 14 alternative movement directions has good search performance in the three-dimensional scene. The results show that the reward function of the proposed Sinfotaxis has good performance compared with other Infotaxis algorithms in both two-dimensional and three-dimensional scenes, indicating that the decision function of Sinfotaxis can reach the balance of exploitation and exploration to a certain extent so that the mobile searcher can locate the release source through fewer steps.

In the process of research, some problems of Infotaxis algorithms are also found. In the context of grid search space, when the mobile searcher adopts mobile strategies with more alternative mobile directions, not all the straight-line distances from alternative mobile directions to mobile tracking devices are exactly the same, and the existence of this difference will cause a difference in the prediction of the reward function of mobile tracking devices in the next step. When applying Infotaxis algorithm in the three-dimensional scene, Poisson distribution is used to estimate the number of particles released by the detected release source, which is not suitable for the case when the release rate of the release source is too small. These are some problems worth solving in future work.

## Figures and Tables

**Table 1:** The average number of search steps.

Algorithm	Infotaxis_4	Infotaxis_8	InfotaxisII_4	InfotaxisII_8	Sinfotaxis_4	Sinfotaxis_8
Average steps	127.95	116.73	126.58	100.10	125.60	105.05

**Table 2:** The total number of search steps.

Mobile strategy	Reward function				
	Infotaxis	InfotaxisII	Sinfotaxis	SinfotaxisII	SinfotaxisIII
6	46	102	62	76	74
14	40	47	31	31	32
26	89	39	18	37	18

**Table 3:** The average number of search steps.

Mobile strategy	Reward function				
	Infotaxis	InfotaxisII	Sinfotaxis	SinfotaxisII	SinfotaxisIII
6	55.67	72.92	39.12	39.53	43.08
14	66.56	86.01	30.40	35.71	45.18
26	67.45	85.89	38.53	47.34	87.69

## Data Availability

The data used to support the findings of this study are included within the article.
